# On the Correlation of Context-Aware Language Models With the Intelligibility of Polish Target Words to Czech Readers

**DOI:** 10.3389/fpsyg.2021.662277

**Published:** 2021-06-30

**Authors:** Klára Jágrová, Michael Hedderich, Marius Mosbach, Tania Avgustinova, Dietrich Klakow

**Affiliations:** ^1^Collaborative Research Center 1102: Information Density and Linguistic Encoding, Saarland University, Saarbrücken, Germany; ^2^Saarland Informatics Campus, Spoken Language Systems, Saarland University, Saarbrücken, Germany; ^3^Language Science and Technology, Saarland University, Saarbrücken, Germany

**Keywords:** intercomprehension, predictive context, Polish, Czech, context-aware language models, Long Short-Term Memory, transformer, surprisal

## Abstract

This contribution seeks to provide a rational probabilistic explanation for the intelligibility of words in a genetically related language that is unknown to the reader, a phenomenon referred to as intercomprehension. In this research domain, linguistic distance, among other factors, was proved to correlate well with the mutual intelligibility of individual words. However, the role of context for the intelligibility of target words in sentences was subject to very few studies. To address this, we analyze data from web-based experiments in which Czech (CS) respondents were asked to translate highly predictable target words at the final position of Polish sentences. We compare correlations of target word intelligibility with data from 3-g language models (LMs) to their correlations with data obtained from context-aware LMs. More specifically, we evaluate two context-aware LM architectures: Long Short-Term Memory (LSTMs) that can, theoretically, take infinitely long-distance dependencies into account and Transformer-based LMs which can access the whole input sequence at the same time. We investigate how their use of context affects surprisal and its correlation with intelligibility.

## 1. Introduction

In the research domain of intercomprehension, the intelligibility of stimuli has been, among other linguistic and extra-linguistic factors, traditionally explained by the linguistic distance of the stimulus toward a language in the linguistic repertoire of the reader, mostly the native language (L1) (e.g., Gooskens, 2007; Möller and Zeevaert, 2015; Golubović, 2016) or a combination of the L1 and other acquired languages (Vanhove, 2014; Vanhove and Berthele, 2015; Jágrová et al., 2017). It has been shown many times that the lower the measurable cross-lingual similarity or regularity of orthographic correspondences (Stenger et al., 2017) is, the more the languages are mutually intelligible in general. This applies to individual words in language pairs, too: The lower the linguistic (orthographic, phonetic, and morphological) distance between a concrete word pair, the more the words are expected to be comprehensible to the reader of the respective other related languages.

So far there have been only a few studies focusing on the role of context as an additional factor influencing the mutual intelligibility of target words. Muikku-Werner (2014) observed that the role of neighborhood density (number of available similar word forms that readers might consider suitable translation equivalents) decreases through context since the potential other options have to fit the syntactic frame. She also found that it appears easier for respondents to guess a frequent collocate of a word, once the other word is successfully recognized (Muikku-Werner, 2014, p. 105). In a study on the disambiguation of false friends with students of Slavic languages, Heinz (2009) points out that the amount of correctly understood context is crucial for the correct recognition of target words. He also refers to the negative role that context can play: Previous (correct) lexical decisions can be revised to formulate an utterance that respondents believe is reasonable.

Jágrová (2018) investigated the influence of divergent word order in Polish (PL) noun phrases (adjective-noun vs. noun-adjective) on their intelligibility to Czech (CS) readers, since the noun-adjective linearization is more typical in PL than in CS which is reflected in higher surprisal scores of the CS translations of the stimuli. She correlated the product of linguistic distance and 3-g language model (LM) surprisal (“overall difficulty”) of the stimuli phrases to processing time and intelligibility and found a higher correlation than with linguistic distance only. This method of determining an overall difficulty consisting of distance and surprisal for individual words within sentences was also applied in Jágrová et al. (2019) in “an attempt to use LMs to describe the role of context in the stimuli and translations thereof” (Jágrová et al., 2019, p. 261), without claiming to present statistically sufficient data for the PL-to-CS scenario (12 sentences, 16 respondent pairs). There it was found that the calculated difficulty levels of the words within the stimuli did not always agree with the actual performance of the respondents. Contrary to the expectations of the authors, even cognates with very low linguistic distance or internationalisms that are identical in both languages were not always translated correctly, especially when they also had low corpus frequency and thus high surprisal scores. Respondents often considered these words unlikely or not fitting the context. In another study by Jágrová and Avgustinova (2019), data from a representative sample of stimuli sentences and respondents was collected in a web-based cloze translation experiment in the same language-reader-scenario. In the present study, we build upon the data from their experiment.

The language models applied in the studies by Jágrová (2018), Jágrová et al. (2019), and Jágrová and Avgustinova (2019) were all 3-g models. The principle according to which these models work is that they iterate through a training corpus and count all occurrences of any three subsequent words. When then applied to a sentence, they can help statistically assess the predictability of a word in relation to its two preceding words. In practice, however, the sentential context relevant for the intelligibility of a target word can be larger than only its two preceding words. Consequently, other types of statistical LMs might be better in capturing the role of semantic primes and concepts that allow for correct associations within the sentences.

To verify this hypothesis, we trained different context-aware LMs on the Czech National Corpus (Křen et al., 2016) and the PolEval 2018 language modeling corpus (Ogrodniczuk and Kobyliński, 2018). We applied these LMs to score the PL stimuli sentences used in the experiment by Jágrová and Avgustinova (2019) and on the closest CS translations thereof. We correlated the surprisal scores of the target words and the whole sentences with target word intelligibility and compared them to the correlations with 3-g surprisal from Jágrová and Avgustinova (2019). Although all correlations proved to be fairly low, we found slightly better results for the target word surprisals from the CS context-aware models. In individual examples, we found that the context-aware models appear to be better suitable to capture the predictability of semantic associations within the sentences, while 3-g models appear to be better representations of predictability caused by collocates directly preceding the target words.

This study is structured as follows. In section 2, we first explain how data from 3-g LMs were correlated with target word intelligibility in Jágrová and Avgustinova (2019). We then outline the hypothesis regarding the better performance of context-aware LMs in comparison to 3-g LMs in section 3 and explain their architectures in section 4. Next, we present the results from the context-aware LMs in section 5 and compare them with the correlations observed in Jágrová and Avgustinova (2019). Finally, we summarize the findings in the discussion in section 6.

## 2. Previous Research

In a previous study, using surprisal estimates from 3-g LMs, Jágrová and Avgustinova (2019) showed that predictability in context contributes to the intelligibility of target words in sentence-final position when compared to the intelligibility of the same words without context. They gathered data from web-based cloze translation experiments for highly predictable target words in 149 PL sentences.

The sentence stimuli presented in the experiment are translations of sentences published in a study by Block and Baldwin (2010) who tested a set of 500 constructed sentences in a cloze completion task. In addition to that, Block and Baldwin (2010) validated the predictability of the target words in their sentences in event-related potential(s) experiments. The study resulted in a dataset of 400 high-constraint, high cloze probability sentences. For the study of Jágrová and Avgustinova (2019), those sentences with the most predictable target words (90–99% cloze probability) were translated into PL and applied in cloze translation experiments. Sentences containing culturally specific context were omitted, which resulted in a set of 149 sentences. The translation into PL was provided by a linguist and professional translator who was instructed to keep the original target words in the last position in the sentences.

These 149 sentences were presented to CS respondents who were asked to guess and translate the PL target words into CS. After having filled out a sociodemographic survey and having provided a self-assessment of language skills, only those respondents were admitted to the experiment who did not indicate any prior knowledge of PL. The PL sentences were presented in seven blocks, each consisting of 17–24 sentences. The order of the sentences within a block was randomized. Data of at least 30 respondents (mean age 25.3) were gathered for each target word. To make sure that respondents indeed read the sentential context, the experiment was designed in a way that respondents initially saw only the first word of the sentence and then were asked to click on it to make the next word appear. In this way, they clicked through the whole sentence till the last word (target word) appeared. After clicking on the target word, the window for entering the translation of the target word appeared. The time limit for entering the translation of the target word was set to 20–30 s, depending on the length of the sentence. The respondents were not informed that the target words are highly predictable.

To obtain a baseline for comparison, the PL target words were also presented without any context and in their base forms to other CS respondents as a cognate guessing task. The majority of the words were more comprehensible within the sentences (68.0% intelligibility) than if presented without context (49.7% intelligibility).

For instance, the PL target word *głosu* “voice [genitive]” in the PL sentence

**Figure 1:**
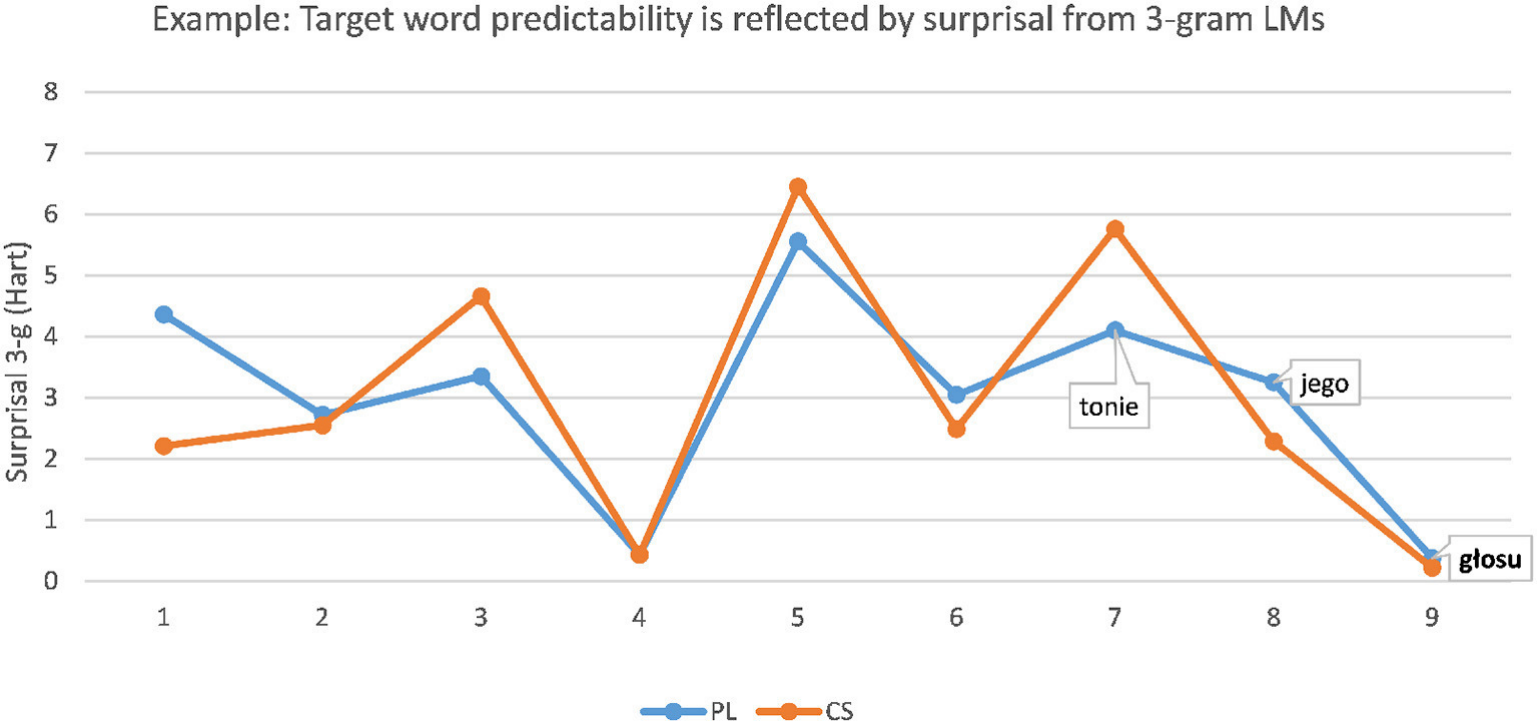
Example: Predictability of PL target word *głosu* “voice [genitive]” is reflected well by the low surprisal score of the target obtained from the 3-g LM.

(1) PL: Ż*e był wściekły, rozpoznała po tonie jego* *****głosu*****.
  (CS: Ž*e byl vzteklý, poznala podle tónu jeho* *****hlasu*****.)
  “That he was mad, she could tell by the tone of his **voice**.”

was translated correctly into CS as a form of *hlas* “voice” more often in the predictive context (93.3%) than without context (26.7%). As shown in Figure 1, the predictability of the target word is, in this case, reflected well by the surprisal scores obtained from the 3-g LM, since PL *głosu* (CS *hlasu*) “voice [genitive]” is highly predictable after PL *tonie jego* (CS *tónu jeho*) “the tone of his.”

The PL 3-g LM was trained on the PL part of InterCorp (Čermák and Rosen, 2012), and the CS LM was trained on the SYN2015 version of the Czech National Corpus (CNC, Křen et al., 2015). Kneser-Ney smoothing (Kneser and Ney, 1995) was applied on both LMs. The PL LM provides the information density profile of the stimuli sentences. To obtain the best possible representation of the comprehension process of a CS reader, the PL stimuli sentences were translated literally into CS before the CS LM was applied for scoring (for detail on the method of Vanhove, 2014; Jágrová and Avgustinova, 2019). The blue graph in Figure 1 represents the surprisal of a PL sentence scored by the PL 3-g LM. The orange graph represents the closest literal CS translation of this PL sentence scored by the CS 3-g LM accordingly.

In other sentences, however, the predictability of the target word resulting in greater ease of understanding was not reflected by the surprisal scores from the PL 3-g LM. For instance, the PL target word *gwoździa* “nail [genitive]” in the sentence

**Figure 2:**
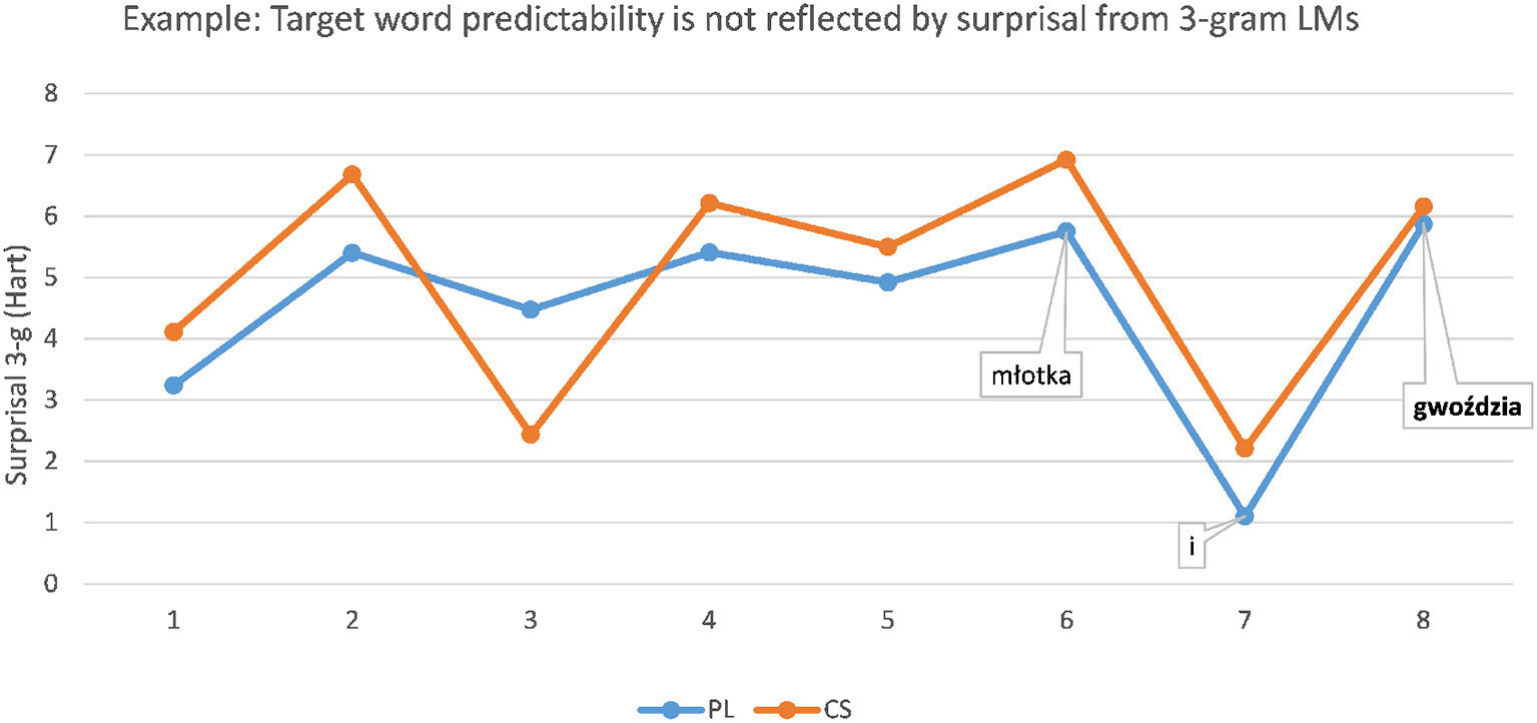
Example: Predictability of PL target word *gwoździa* “nail [genitive]” is not reflected well by the 3-g language model (LM): surprisal curve rises at the target word.

(2) PL: *Aby zawiesić obraz Ted potrzebował młotka i* *****gwoździa*****.
  (CS: *Aby zavěsil obraz, Ted potřeboval kladivo a* *****hřebík*****.)
  “To hang the picture Ted needed a hammer and a **nail**.” (Block and Baldwin, 2010)

was translated more often correctly as a form of CS *hřeb*í*k* “nail” in context (53.3%) than without context (3.03%). However, as shown in Figure 2, the 3-g LM displays a rise in surprisal at the target word position, which is a typical indication of high processing difficulty due to unexpectedness in context. This suggests that the predictability of the target word does not depend exclusively on the immediately preceding words, as could have been reflected by the 3-g LM. Instead, the better comprehension of the target seems to be connected to the correct identification of the concept of hanging a picture: PL *zawiesić* “to hang” is a cognate of CS *zavěsit*, the sentence-initial conjunction *aby* “to” as well as the noun *obraz* “picture” are identical in form and meaning in both languages, PL *potrzebował* “he needed” is a cognate of its CS translation *potřeboval*. PL *młotka* “hammer [genitive]” preceding the target word is a non-cognate to its CS translation equivalent *kladivo*. However, there might be a clue in the CS lexicon through the concept of *mlátit* “to hit” or *mlat* as in *sekeromlat* “threshel, stone axe,” provided that the CS respondents successfully apply the regular PL:CS correspondence ł*o*:*la/lá* in the stem.

Even though the context was helpful for the comprehension of targets in most of the sentences, the situation was reversed for some target words in context if compared to the condition without context. An analysis of the errors made by respondents revealed some systematic patterns, such as L1 interferences, inferences from other acquired languages, or perceived morphological mismatches. Also, priming by readers or association with a dominant but misleading concept in the sentence seems to have played a crucial role in the misinterpretations of some target words. For instance, the PL target word *dzień* (CS *den*) “day” in the sentence

(3) PL: *Dentysta zaleca myć zȩby dwa razy na* *****dzień*****.
  (CS: *Zubař doporučuje čistit si zuby dvakrát za* *****den*****).
  “The dentist recommends brushing your teeth twice a **day**.” (Block and Baldwin, 2010)

was translated wrongly by some respondents as *dáseň* “gum.” Not only are PL *dzień* and CS *dáseň* orthographically relatively similar [Levenshtein distance: 0.5 (Levenshtein, 1966), the mean pronunciation-based orthographic distance of the 149 target words is 42.6%], but also does the concept of the easily identifiable PL *dentysta* (CS *dentista* or *zubař*) “dentist” mislead respondents to an association of the target word with the dentist. The intelligibility of PL *dzień* for CS respondents was higher without context (80.0%) than in context (66.7%). The question is whether such effects can be predicted by an LM that would also take into account cross-lingual similarity. We explore this setting in section 4.6.

It has to be mentioned that sentence context is not equally easy to understand in all test sentences, some of the sentences contain non-cognates or false friends, while others do not. Also, the orthographic distance of cognates is different in each sentence. Admittedly, it is difficult to capture the whole complexity of intercomprehension in these translation experiments and to control for a whole range of (linguistic) factors that come into play when the context is concerned.

In an ideal world, all words of which the stimuli sentences consist should have been tested for intelligibility separately to reliably assess how much of the context the respondents understand. Although it was not tested how intelligible the context is, it was approximated by measuring the linguistic distance (lexical and orthographic distance) of the stimuli sentences toward the closest CS translation in Jágrová and Avgustinova (2019). The distance of the target word and the total number of non-cognates per sentence were then added as variables into a multiple linear regression model and could, together with the sum of surprisal of the PL sentence, account for 49.6% of the variance in the data (Jágrová and Avgustinova, 2019, p. 15).

Jágrová and Avgustinova (2019) also found that besides high correlations with orthographic distance (*r* = −0.772, *p* < 0.001 without context and *r* = −0.680, *p* < 0.001 in predictive context), the correlation of intelligibility with surprisal depends on the lexical similarity of the target words. For the whole set of 149 sentences, the best correlation found was a fairly low one with the sum of surprisal of the whole PL sentence (*r* = −0.215, *p* < 0.01). When excluding sentences with target cognates (words with etymologically related translation equivalents in both languages) from the analysis, the correlation of intelligibility with the total surprisal of the PL sentence reaches *r* = −0.411, *p* < 0.01. Three-gram surprisal and intelligibility correlate best for sentences in which the target words are false friends (*r* = −0.443, *p* < 0.01), especially those that, despite their misleading character, allow for correct semantic associations with the correct translation. Even though all correlations turned out relatively low, predictability effects and associations seem to be more important for targets with high linguistic distance, especially for non-cognates and false friends (lexical distance), than for cognates with low linguistic distance. For instance, PL *drzewo* was translated more often correctly as CS *strom* “tree” (36.6%) or *rodokmen* “family tree” in the sentence

(4) PL: *Aby dowiedzieć siȩ czegoś o swoich przodkach, narysowali genealogiczne* *****drzewo*****.
  (CS: *Aby se dozvěděli něco o svých předc*í*ch, nakreslili genealogický* *****strom***** / *****rodokmen*****.)
  “To learn about their ancestors they drew a family **tree**.” (Block and Baldwin, 2010)

than in the condition without context (0%). There it was frequently mistaken for its CS false friend *dřevo* “wood.” Together with the partly identifiable context of this sentence [PL *dowiedzieć siȩ* (CS *dozvědět se*) “to learn (about)”; PL *o swoich przodkach* (CS *o svých předc*í*ch*) “about their ancestors”], PL *drzewo* allows for a correct semantic association of wood and trees (Jágrová and Avgustinova, 2019, p. 11).

## 3. Hypothesis

Since the 3-g LM used by Jágrová and Avgustinova (2019) cannot reflect the influence of contextual cues from any other position in the sentence than the two words immediately preceding the target word, we hypothesize that the intelligibility of highly predictable target words will have a stronger correlation with surprisal values obtained from language models which incorporate information from the entire sentence than with surprisal values from 3-g LMs.

## 4. Methods

We build upon the study by Jágrová and Avgustinova (2019) and estimate the surprisal of target words in a given sentence by relying on language models that are capable of considering context beyond 3-g.

In recent years, two main approaches have dominated context-aware, neural LMs: Long Short-Term Memory (LSTM) and more recently Transformer. For both architectures, we investigate whether their use of sentence-level context affects surprisal and its correlation with intelligibility. We start by providing a brief recap on (neural) language modeling.

### 4.1. Language Modeling

Language models are machine learning models that are typically trained on text corpora and can predict the probability of a word given its context. As an example, an LM trained on a standard English corpus, given the start of the sentence *A small, green* would assign most likely the word *frog* a higher probability for continuing the sentence than the word *cow*. The probability for a target word, given its context, is obtained *via* a learned model that bases its predictions on occurrence statistics in the training corpus.

Most commonly, an LM predicts the probability of a word given the previous (left) context. Formally, for a sentence *s* consisting of words or tokens *w*_1_, …, *w*_*n*_, an LM computes the probability *p*(*w*_*t*_|*w*_*t*−1_, …, *w*_0_). The probability of a sentence can be obtained by factorizing the joint probability as a product of conditional probabilities, i.e., by applying the product rule of probabilities:

(1) $$\begin{array}{l} p\left(s\right)=\overset{n}{\underset{t=1}{\prod }}p\left(w_{t}|w_{t-1},...,w_{0}\right) \end{array}$$

Traditionally, count-based n-gram models have been used for language modeling. In this case, the previous context is limited to *n* − 1 words. A 3-g model, therefore, can only compute the probability of a word given its two predecessors, i.e., *p*(*w*_*t*_|*w*_*t*−1_, *w*_*t*−2_). Increasing the value of *n* for count-based models is difficult due to factors like data sparsity (Jelinek and Mercer, 1980).

### 4.2. Long Short-Term Memory

Long Short-Term Memories (Hochreiter and Schmidhuber, 1997) are a form of Recurrent Neural Networks (RNNs) (Elman, 1990). They learn a parametric model of the distribution of words given their context. These machine learning models can handle sequences of input words of arbitrary length. This removes the hard limitation of history size *n* that *n*-gram models have. At each time-step *t*, the RNN obtains as input the previous word or token *w*_*t*−1_. It then updates its internal state based on that input and its previous internal state. As output, at each time-step, the probability for the current word is given *p*(*w*_*t*_|*w*_*t*−1_, …, *w*_0_).

While RNNs have in theory no limitation on sequence length, in practice, effects like vanishing gradients (Bengio et al., 1994) do limit the amount of previous words that are taken into consideration for the probability of the next token. LSTMs contain special components, such as cell states that improve the handling of such long-term dependencies. An in-depth discussion of the use of LSTMs for language modeling is given in Sundermeyer et al. (2012).

In this study, we build a four layer LSTM with embedding and hidden state sizes of 300. Dropout (Srivastava et al., 2014) of 0.1 is applied between the layers, and gradient clipping is performed with a gradient norm size of 1. As an optimizer, we use Adam (Kingma and Ba, 2015) with a learning rate of 2.5 * 10^−4^.

### 4.3. Transformer

Originally proposed for the task of neural machine translation, Transformers (Vaswani et al., 2017) have recently shown strong empirical performance on various natural language processing tasks and have become the predominant architecture for many natural language processing tasks. Other than RNNs, such as LSTMs, Transformers typically do not contain any recurrence and hence have access to the whole input sequence at once *via* an attention mechanism. They can model *p*(*w*_*t*_|*w*_*t*−1_, …, *w*_0_) while taking into consideration all previous context words *w*_*t*−1_, …, *w*_0_ in equal measure. This allows them to make more efficient use of context. Given a large enough input size and positional encodings, Transformers have become the dominating architecture for neural language modeling (Al-Rfou et al., 2019; Dai et al., 2019).

In this study, we train two different Transformer based LMs: (1) a vanilla Transformer decoder with 16 hidden layers, learned positional encoding and a context size of 32 tokens (Al-Rfou et al., 2019) and (2) a 16-layer Transformer-XL decoder with relative positional encodings (Dai et al., 2019). The same gradient clipping and optimizer are used for the LSTM. We choose a context size of 32 tokens based on the sentence length statistics of the stimuli sentences.

### 4.4. Corpora

The PL LMs were trained on the PolEval 2018 language modeling corpus (Ogrodniczuk and Kobyliński, 2018). It contains 20 million sentences selected from PL Wikipedia, Internet forums, PL books, the National Corpus of Polish Przepiórkowski et al. (2012), and the Polish Parliament Corpus (Ogrodniczuk, 2012). We used the unsegmented version released by the PolEval organizers^1^. This corpus is larger than the Polish part of InterCorp (Čermák and Rosen, 2012) used by Jágrová and Avgustinova (2019).

The CS LMs were trained on the SYN v4 version of the Czech National Corpus (Křen et al., 2016), a collection of contemporary written CS containing ~4.3 billion tokens. This is the same data as in the study by Jágrová and Avgustinova (2019).

We tokenized both corpora using byte-pair-encoding (Sennrich et al., 2016) and using the SentencePiece toolkit (Kudo and Richardson, 2018). More specifically, for each of the corpora, we automatically create a vocabulary containing the 32.000 most frequent subunits (so-called subwords) and then tokenize the training data as well as the stimuli sentences according to this vocabulary. Both LSTM and Transformer models use the same vocabulary. If a target word is tokenized into several subunits, the probability of the target word is the product of the probabilities of the subunits.

The PL stimuli sentences were scored with the LMs trained on the PL corpus. To obtain the surprisal scores for the CS versions of the sentences and hence to represent their understanding by the CS reader, both the closest CS translation (not necessarily grammatically correct) and a grammatically correct CS translation were scored by the LMs trained on the CS corpus. Models of both languages were used to find out if the surprisal of the stimulus (PL) or the language of the readers (CS) correlates better with target word intelligibility.

### 4.5. Language Model Performance

The performance of LMs is commonly measured in perplexity over the test corpus *T*. It is defined as

(2) $$\begin{array}{l} PPL\left(T\right)=2^{-\sum_{w\in T}p\left(w\right)\log_{e}p\left(w\right)} \end{array}$$

where *w* are all the words or subwords in *T*. The lower the perplexity of the LM, the better is the performance of the model on predicting the correct next token. The test perplexities for the CS and PL language models are given in Tables 1, 2, respectively. For both languages, the Transformer model outperforms the LSTM and Transformer XL performs best. To the best of our knowledge, we reach a new state-of-the-art for language modeling on the PL corpus (Czapla et al., 2018).

**Table 1:** The perplexity of the language models on the CS validation corpus.

**Model**	**Subword PPL**	**Word PPL**
LSTM	17.85	38.80
Transformer	15.59	32.67
TransformerXL	**13.94**	**28.35**

*The lowest perplexity values are marked bold*.

**Table 2:** The perplexity of the LMs on the PL validation corpus.

**Model**	**Subword PPL**	**Word PPL**
LSTM	49.83	125.5
Transformer	31.12	70.11
TransformerXL	**29.92**	**66.78**
ULMFiT-SP (Czapla et al., 2018)	–	117.67
ULMFiT-SP (Czapla et al., 2018)	–	95.0

*The lowest perplexity values are marked bold*.

### 4.6. Toward a Model of the Reader

Following the previous study, we train the aforementioned LMs on PL and CS and then evaluate their surprisal on sentences in the same language. In addition, we also propose a model that is conceptually closer to the human participants. In this case, these are CS native speakers who read PL text. We, therefore, also use the CS Transformer LM to compute surprisal on PL sentences. The model should, e.g., have a low surprisal by the PL word *testamencie* “testament [locative]” as it is close to its CS translation *testamentu*. This is in contrast to the PL word *gwoździa* “nail [genitive]” where the equivalent in CS would be a form of *hřeb*í*k* which should result in a high surprisal for the model. It is important to note that this is possible since the surprisal of the Transformer model is computed on a subword or character level (as shown in section 4.4) and not exclusively on a word level. While the PL word *testamencie* will most likely be unknown to a CS LM, its subwords *te, sta, men*, and *cie* are part of the subword vocabulary of the model.

There are several PL characters with diacritics, e.g., *a̧*, ć, and ł, that are not part of the CS alphabet and thus unknown to this LM. As an attempt to overcome this issue, such PL characters are mapped to CS characters that CS respondents assumed to be corresponding in a previous experiment. There, CS respondents were asked to read out PL stimuli including the unknown PL characters aloud and translate them (Jágrová, 2021). With the help of the transcripts of these recordings, it was possible to obtain statistics about how likely an unknown character was pronounced similar to a (seemingly) corresponding CS character. We use these insights to map certain PL characters to the CS alphabet. In the case of PL ł, for instance, the CS character *l* would also be the linguistically correct correspondence. However, while the linguistically correct CS correspondence for PL ć would be *t* (regular correspondence in infinitive endings, which a CS reader is not expected to be aware of), we map it to CS č.

### 4.7. Intelligibility

The intelligibility of the word is measured here as the percentage of correct translations provided by respondents for this word. For instance, if the PL word *dzień* “day” has intelligibility of 80%, it means that 80% of the CS respondents translated the word correctly. As for the scoring of responses of the participants, the experiment software automatically classified responses as correct or wrong, according to the previous definition. All responses were, however, manually checked afterward so that cases which were classified as wrong but where respondents had understood the stimulus, e.g., typos, missing letters at word end due to time restrictions, or synonyms, could be categorized as correct subsequently. Also, responses that were base forms of targets were counted as correct even if the target word was inflected. The wrong gender of verb forms was tolerated if the translation was otherwise correct, but the wrong tense was not accepted.

### 4.8. Predictors

We first perform a linear regression with surprisal as the main predictor in question and then add other predictors into a multiple linear regression model. Surprisal as a predictor variable is provided by the models in the unit *nat*. For each sentence and each model trained, we determine the surprisal of the target word as well as the surprisal of the whole sentence. Since higher surprisal is related to higher difficulty, higher surprisal should predict lower intelligibility of an item. If a word is segmented into subword units, then its surprisal is the product of the subword surprisals.

As a representation of the (dis-)similarity of the PL stimulus toward CS, a measure referred to as total pronunciation-based distance is determined for the whole sentence, the final 3-g, 2-g, and target word and examined for correlations with intelligibility. The distances are calculated automatically with the help of the *incom.py* toolbox (Mosbach et al., 2019) for each word. Distances of the 2-g, 3-g, and sentences are the mean distances of the individual words they consist of. For the calculation, two words are aligned by their consonants and vowels in a way that the cheapest alignment option is preferred. The alignment cost for every single PL:CS character pair can be defined when using the *incom.py* tool. For this purpose, a cost of 1 is charged for every different character. As illustrated in Table 3, the pronunciation-based distance differs from traditionally calculated Levenshtein distance (Levenshtein, 1966) in a way that it does not charge any costs for the alignment of such characters whose pronunciation should be obvious to the respondents: *y*:*i, i*:*y*, ł:*l, w*:*v*, ż:ž (PL:CS). The share of different characters is normalized by the alignment length of the word pair and given as a percentage. The more distant a PL word, the less it is expected to be intelligible to the CS respondents. The total pronunciation-based distance measure also incorporates lexical distance by assigning a distance of 100% to non-cognates.

**Table 3:** Traditionally calculated Levenshtein distance vs. pronunciation-based distance.

**Traditional Levenshtein distance**										
	1	2	3	4	5	6	7	8	9	10
PL			s	i	ł	o	w	n	i	ȩ
CS	p	o	s	i	l	o	v	n	u	
Distance	1	1	0	0	0.5	0	1	0	1	1
Normalized distance	55%									
**Pronunciation-based Levenshtein distance**										
PL			s	i	ł	o	w	n	i	ȩ
CS	p	o	s	i	l	o	v	n	u	
Distance	1	1	0	0	0	0	0	0	1	1
Normalized distance	40%									

The total number and the percentage of non-cognates per sentence are determined as an additional separate predictor of lexical (dis-)similarity. For this purpose, non-cognates are PL words that, in the given context, do not have a CS translation equivalent with the same or a related root in terms of etymological origin. For instance, the sentence in example (2) contains one non-cognate, *gwoździa* “nail [genitive].” The other seven words of the sentence are cognates. If normalized by the number of words in the sentence, the percentage of non-cognates in this sentence is 12.5%. The more non-cognates a CS respondent encounters in a sentence, the less intelligible the sentence should be.

## 5. Results

### 5.1. Regression Results

**Figure 3:**
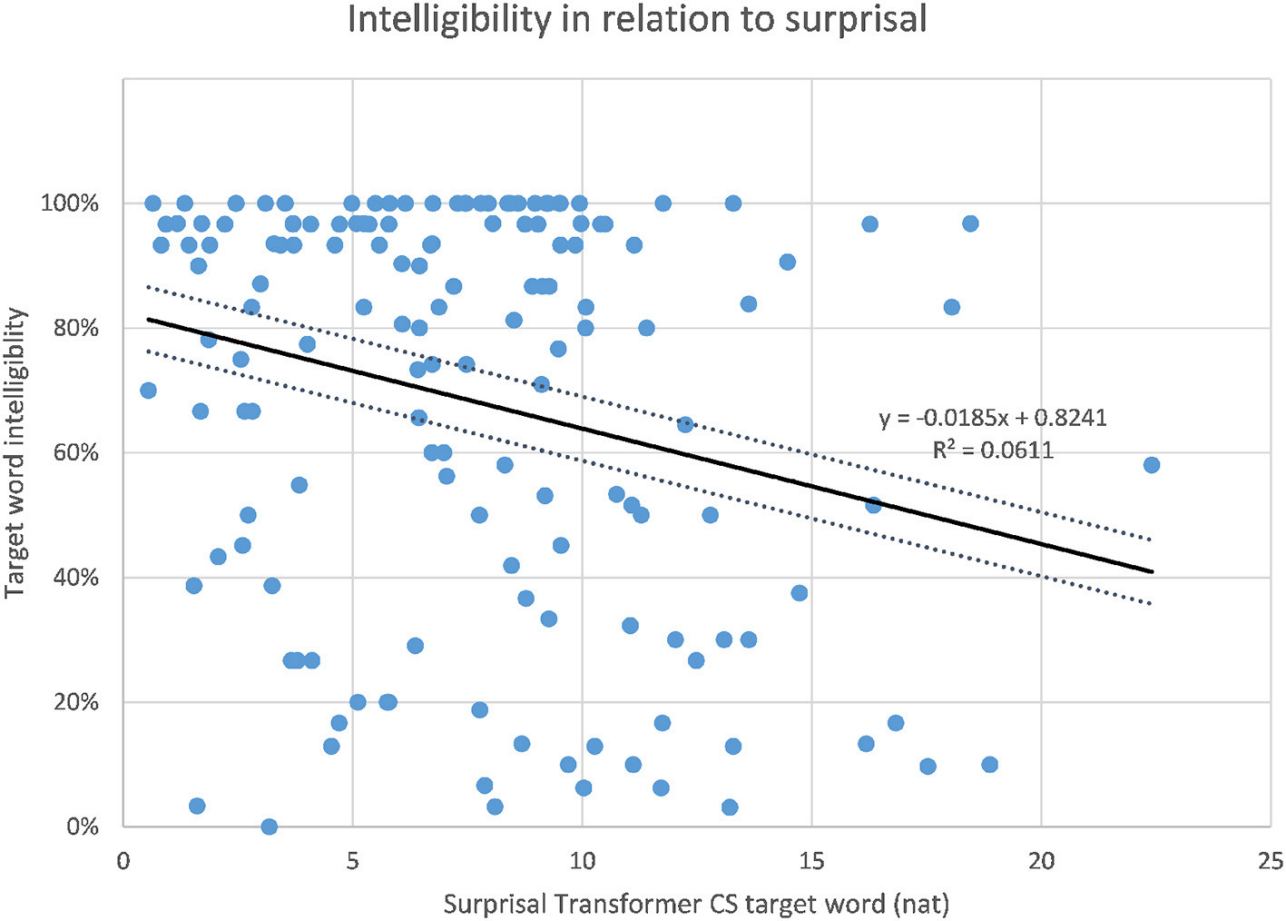
Intelligibility of target words and surprisal from the CS Transformer model.

**Figure 4:**
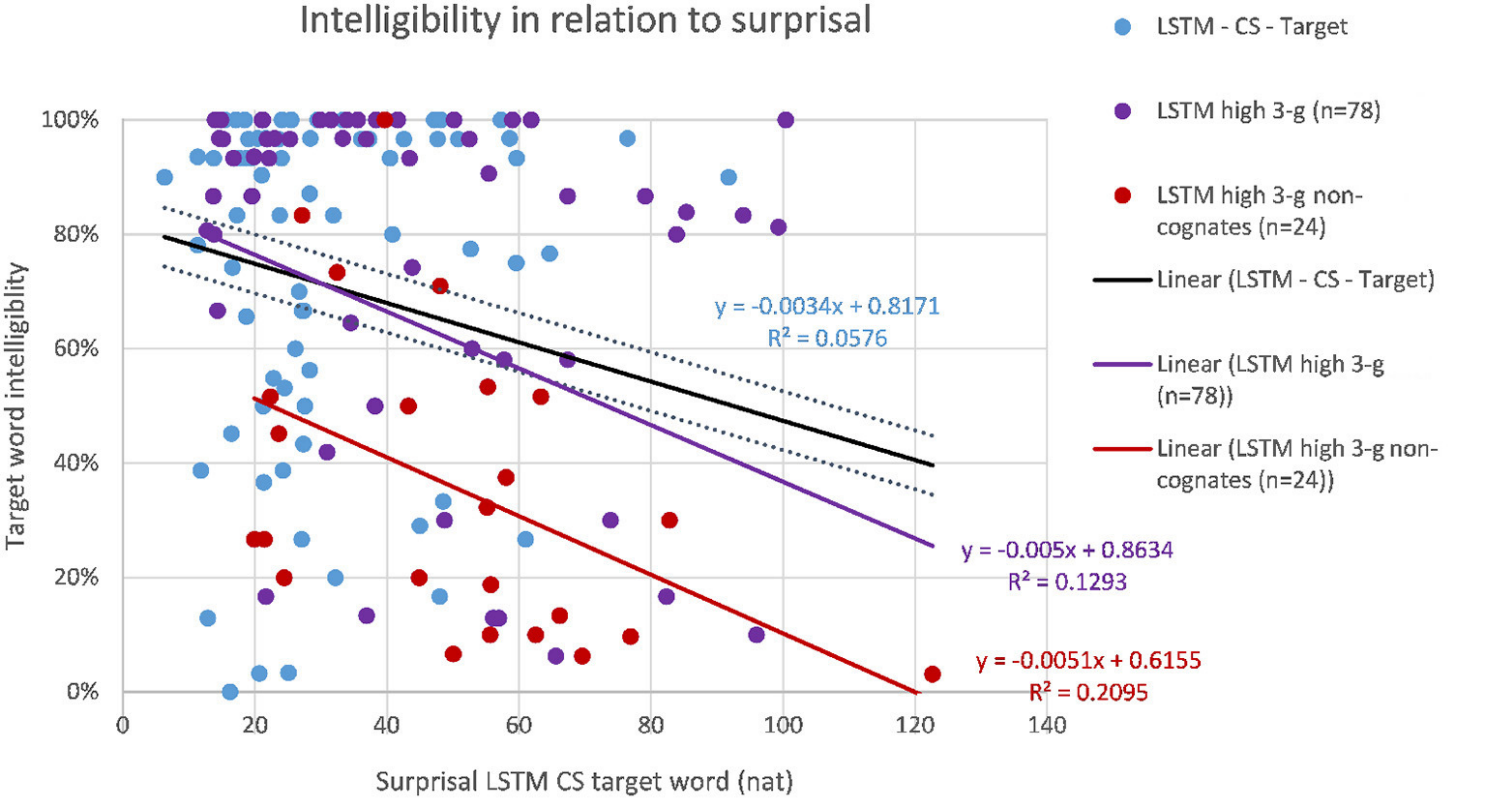
Intelligibility of target words (including filtered subsets) and surprisal from the CS LSTM model.

**Figure 5:**
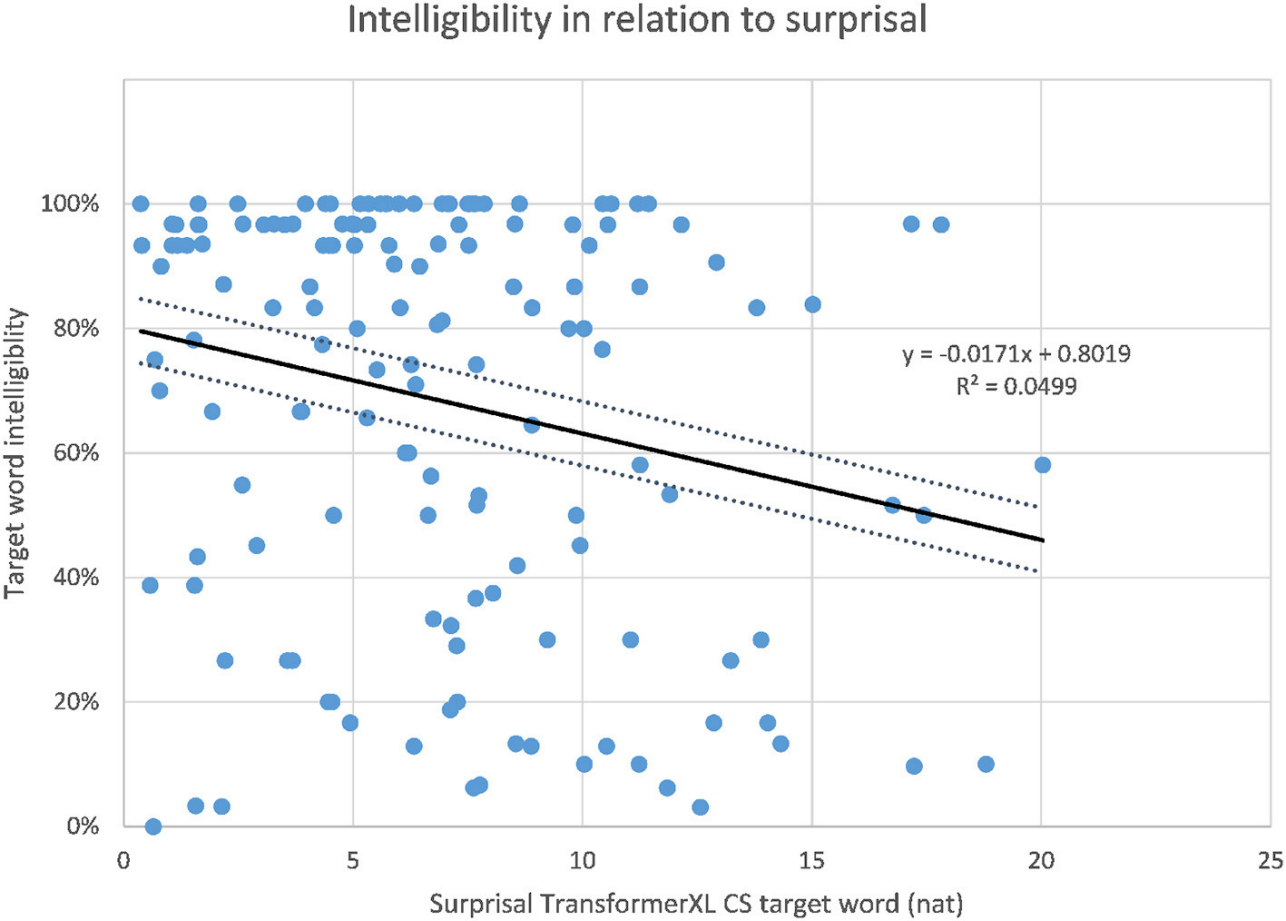
Intelligibility of target words and surprisal from the CS Transformer XL model.

The correlations of target word intelligibility and surprisal from the context-aware LMs are listed in Table 4 together with the correlations of the 3-g surprisal from the previous study for comparison. When considering the whole dataset of 149 sentences, the highest correlation of intelligibility and surprisal could be found for the target word surprisal from the CS Transformer model (*r* = −0.247, *p* < 0.01, as shown in Figure 3), followed by the CS LSTM (Figure 4), and the CS Transformer XL (Figure 5). Thus, the surprisal from all three models correlates slightly stronger with intelligibility than the surprisal from the 3-g models in the previous study, which weakly confirms the hypothesis. No significant correlation could be found with the sum of surprisal per sentence or with the surprisal obtained from the PL versions of the context-aware models.

**Table 4:** Correlations of the context-aware LMs with intelligibility (all sentences).

**Model**	**Surprisal of**	**Correlation**
Transformer CS	target word	*r* = −0.247, *p* < 0.01
LSTM CS	target word	*r* = −0.240, *p* < 0.01
TransformerXL CS	target word	*r* = −0.223, *p* < 0.01
3-g PL (Jágrová and Avgustinova, 2019)	sentence (sum)	*r* = −0.215, *p* < 0.01
Reader Model	target word	*r* = −0.214, *p* < 0.01
3-g CS (Jágrová and Avgustinova, 2019)	target word	*r* = −0.191, *p* < 0.05
3-g PL (Jágrová and Avgustinova, 2019)	target word	*r* = −0.186, *p* < 0.05
TransformerXL PL	target word	*r* = −0.150, *p*>0.05
LSTM PL	target word	*r* = −0.148, *p*>0.05
Transformer PL	target word	*r* = −0.141, *p*>0.05

**Figure 6:**
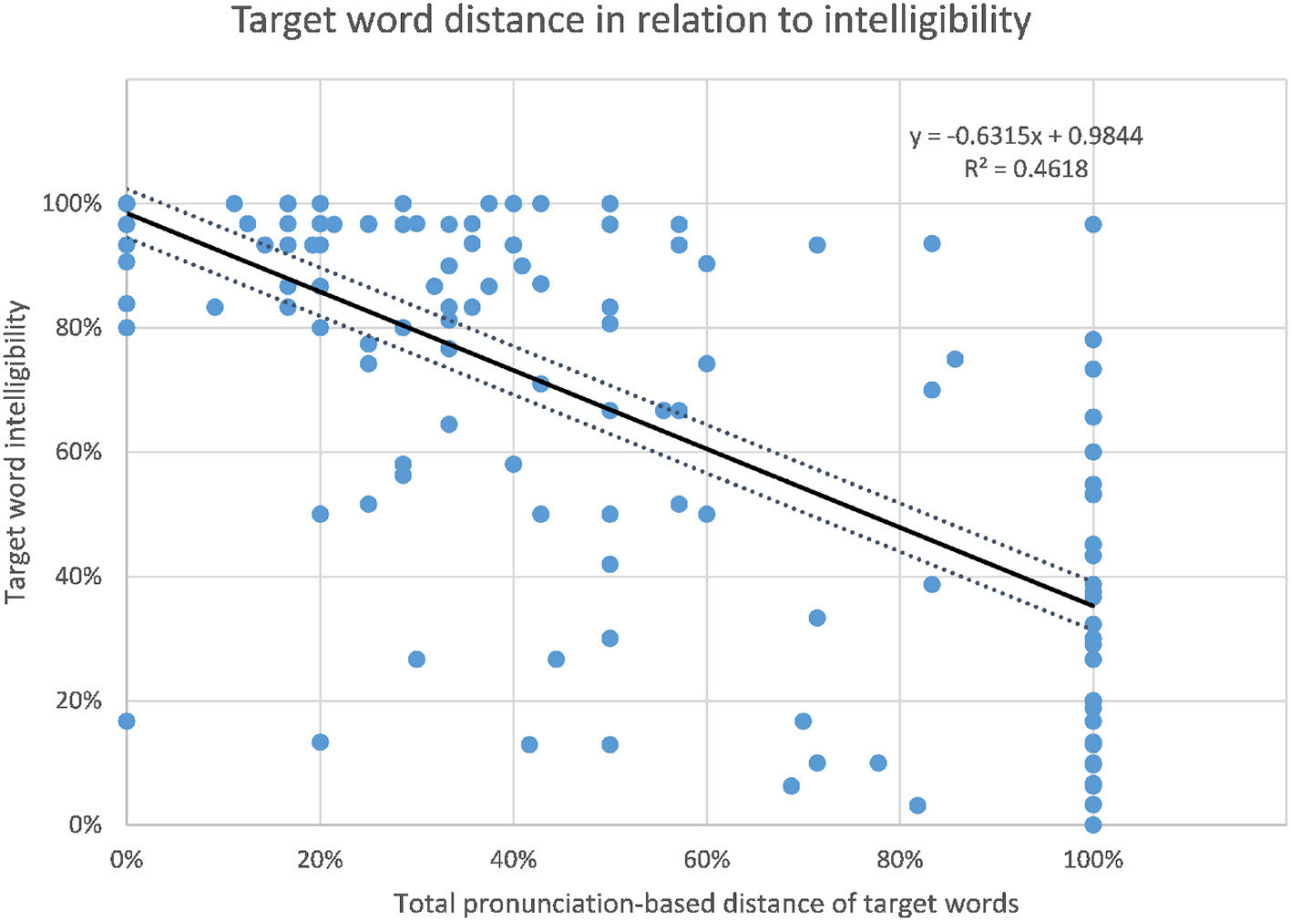
Relation of target word intelligibility and target word distance.

**Figure 7:**
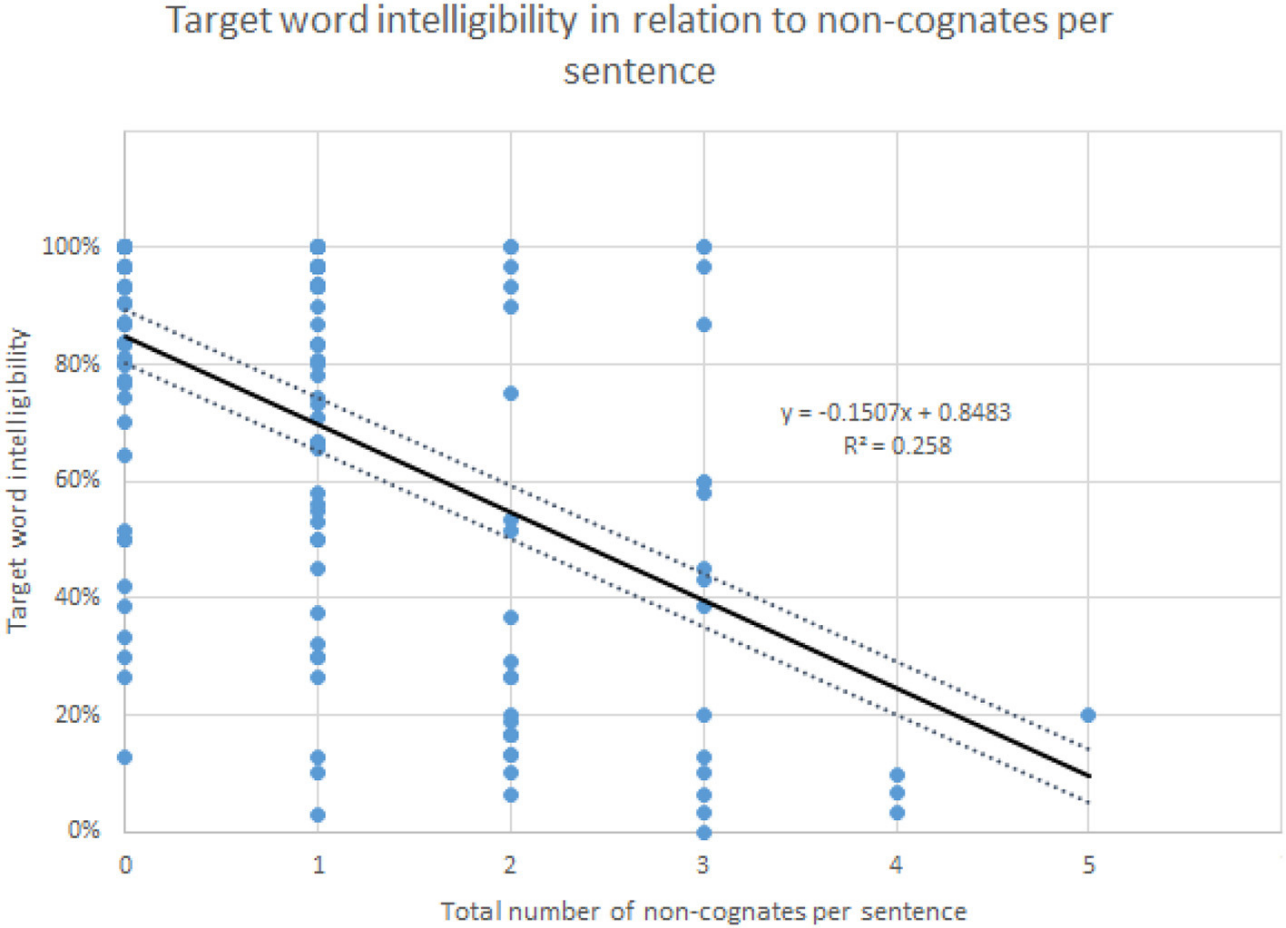
Relation of target word intelligibility and the number of non-cognates per sentence.

As pointed out in the introduction, the intelligibility scores have high (negative) correlations with linguistic distance. The best correlation found regarding distance is that of target word distance and intelligibility. Intelligibility and target word distance correlate with *r* = −0.680, *p* < 0.01 (Jágrová and Avgustinova, 2019, p. 15), as shown in Figure 6. As presented in Figure 7, the number of non-cognates per sentence as a measure of lexical distance also shows a significant negative correlation with target word intelligibility with *r* = −0.507, *p* < 0.0001, although the correlation is lower than that of target word distance.

### 5.2. Multiple Linear Regression Analyses

When we add the variables surprisal and linguistic distance into a multiple linear regression model, the best fitting model is one that consists of the pronunciation-based distance of the target word, the total number of non-cognates per sentence, and surprisal from the CS Transformer model. A regression equation was found [*F*_(3, 145)_ = 59.569, *p* < 0.0001] with an adjusted *R*^2^ of 0.543, *p* < 0.001. This is higher than the *R*^2^ = 0.496, *p* < 0.001 of the model containing 3-g surprisal reported in Jágrová and Avgustinova (2019). The predicted intelligibility of the target word is equal to 1.209 − 0.648 * *distance* − 0.065 * *NC* − 0.023 * *surpTransCS*, where *distance* (in %) is the pronunciation-based distance of the target word normalized by the alignment length of the word pair, *NC* is the number of non-cognates per sentence as a total number (not normalized by the number of words per sentence) and surprisal is measured in *nat* (*surpTransCS* is surprisal from the CS Transformer model). According to the model, the predicted intelligibility of a target word decreased by 0.648% for each % of the distance of the target. As of the model, target word intelligibility decreased by 6.5% for each non-cognate per sentence. For each *nat* of surprisal, target word intelligibility decreased by 2.3%. All three variables distance of the target, number of non-cognates per sentence, and surprisal from the CS Transformer model were significant predictors of target word intelligibility.

### 5.3. Illustrative Examples

For all example sentences mentioned so far, the surprisal scores from the 3-g LMs (target word surprisal PL and CS and sum of surprisal of the PL sentence) are compared to the target word surprisals from the best performing context-aware LMs in Table 5. Contrary to expectations, all models display relatively high coefficients of variance when it comes to target word surprisal of the whole data set, while the coefficient of variance of the 3-g surprisal of the PL sentence is less than half as high. If the LMs provided optimal representations of predictable target words, then target word surprisal would be rather constantly low and would not vary to a high degree.

**Table 5:** Surprisal scores: 3-g vs. context-aware LMs of example sentences 1–7 (surprisal below the mean of the whole dataset is marked bold).

**Example**	**Target**	**3-g**	**3-g**	**3-g PL**	**LSTM**	**Trans**	**TransXL**
		**PL**	**CS**	**sentence**	**CS**	**CS**	**CS**
1	*głosu*	**038**	**0.22**	26.76	40.47	**0.82**	**0.40**
2	*gwoździa*	5.88	6.16	36.19	55.28	10.75	11.90
3	*dzień*	**0.75**	**3.63**	**21.78**	**27.17**	**2.81**	**3.88**
4	*drzewo*	**1.61**	**0.44**	29.28	**21.38**	8.77	7.67
5	*krowy*	3.86	4.05	30.42	**23.03**	**3.70**	**1.06**
6	*sztuki*	3.52	**2.34**	29.03	**22.84**	**3.83**	**2.59**
7	*siłowniȩ*	4.15	5.58	26.52	57.75	8.31	11.25
Mean surp all		3.14	3.85	24.74	38.97	7.59	6.94
SE all		1.76	1.96	5.91	22.73	4.34	4.25
CV (%)		56.12	50.96	23.89	58.34	57.12	61.29

Since it is interesting to observe whether the context-aware LMs perform better with sentences containing semantic associations or hyponymy outside of the final 3-g, which the 3-g LMs were not able to capture, we take a closer look at the results for the following sentences (also listed in Table 5):

(5) PL: *Farmer spȩdził ranek doja̧c swoje* *****krowy*****.
  (CS: *Farmář strávil ráno t*í*m, že dojil svoje* *****krávy*****.)
  “The farmer spent^2^ the morning milking his **cows**.” (Block and Baldwin, 2010)
(6) PL: *Ellen lubi poezjȩ, malarstwo i inne formy* *****sztuki*****.
  (CS: *Ellen má ráda poezii, mal*íř*stv*í *a jiné formy* *****umění*****.)
  “Ellen enjoys poetry, painting, and other forms of **art**.” (Block and Baldwin, 2010)
(7) PL: *Sportowiec lubi chodzić na podnoszenie ciȩżarów na* *****siłowniȩ*****.
  (CS: *Sportovec rád chod*í *na vzp*í*rán*í *do* *****posilovny*****.)
  “The sportsman likes to do weightlifting at the **gym**.” (Block and Baldwin, 2010)

The mean surprisal scores, their SEs, and coefficients of variance for all sentences (*n* = 149) are indicated at the bottom of Table 5 for the different models. All surprisal scores below the mean of the whole dataset (i.e., low surprisal) are marked in bold font. Note that the surprisal for the 3-g models is given in *Hart* (log base 10) while our models use the unit *nat* (log base *e*). While they are not directly comparable, their correlations with intelligibility and the difference to the means for the same models can be compared.

As mentioned earlier, the predictability of the target word *głosu* in example (1) was already reflected well by the 3-g LMs and is also reflected well by the surprisal from the Transformer and Transformer XL model, but surprisingly not by the LSTM. Also, all models assigned a low surprisal to the target *dzień* in example (3), suggesting greater ease of cognitive processing, although its intelligibility was lower in context than without any context. We can observe that the predictability of the target words in examples (5) and (6) is better reflected by the context-aware LMs when compared to the 3-g LMs since their target word surprisals are considerably below average. This suggests that the context-aware models can capture the implication (farmer and cows) in example (5) or the relation of art with poetry and painting in example (6). In the case of example (5) it is likely that the high surprisal score of the 3-g LMs is due to the low corpus frequency of the present participle form *doja̧c* “milking” (as opposed to the more frequent infinitive *doić* “to milk”). In this study, respondents can in the first place rely on target word similarity: PL *krowy* and CS *krávy* “cows” are cognates with a pronunciation-based distance of only 20%. In example (6), however, a correct response can be based only on expectations, since the target word *sztuki* “art [genitive]” is a non-cognate to CS *uměn*í. The remaining sentence in example (6) consists of cognates and should thus be understandable. A possible inference might be drawn through š*tyk* as it occurs in the CS compound and Germanism *majstrštyk* “masterpiece” (or through knowledge of German) which might in addition to the context evoke an association with the concept of art and hence lead the respondent toward a correct understanding of the target.

However, all of the models assigned a relatively high surprisal to the target word *siłowniȩ* in example (7) and *gwoździa* “nail [genitive]” in example (2). In example (2), this might be because PL *gwoździa* and forms of its CS translation equivalent *hřeb*í*k* have very low corpus frequencies in general. It could have been expected that the occurrence of the words for *hanging* and *picture* might lead to the predictability of the context-aware models and hence lower surprisal of *hammer and nail*, but, judging from the surprisal scores, these concepts most likely do not co-occur often enough in the training corpora. As for what could be expected regarding the transformation of the target word *gwoździa* by the reader model (section 4.6), the model transforms PL ź into CS ž, resulting in *gwoždzia*, which is then scored by the CS model. Since this string of characters is rather unusual in CS, it is no surprise that the surprisal score from this model is rather high. Accordingly, PL ł in the preceding collocate *młotka* “hammer [genitive]” is transformed into CS *l* and not into the linguistically correctly corresponding root *mlat* or *mlát*, so that *mlotka* is scored by the model. Since this is a non-word in CS, it is unlikely to lead to a lower surprisal of *gwoždzia*.

Despite its high surprisal score, the target word *siłowniȩ* “gym” was translated more often correctly as a form of CS *posilovna* in context (58.1%) than without context (30.3%). The whole sentence should be more or less understandable for the CS respondents: PL *sportowiec* “sportsman” is an orthographically relatively close cognate to CS *sportovec*, PL *lubi* “likes” can be inferred from CS *l*í*bit* (*se*) “to like [reflexive],” PL *chodzić* “to go” through CS *chodit*, the preposition *na* is, in this case, identical in form and meaning in both languages, PL *podnoszenie* “lifting” can be segmented into the prefix *pod* “under,” which is again identical in both languages, and *noszenie* which is related to CS *nošen*í “carrying.” The only problem here could be in PL *ciȩżarów* “weights [genitive plural]”: Although it contains the Pan-Slavic root *ciȩż*, which linguistically corresponds to the CS root *těž*, a non-linguist respondent cannot be expected to know of the applicable regular cross-lingual correspondence of *ciȩ*:*tě* (PL:CS). While CS uses the term *vzp*í*rán*í “weightlifting,” PL uses the noun phrase *podnoszenie ciȩżarów* (literally *lifting of weights*) in which *podnoszenie* is post-modified with the genitive plural *ciȩżarów*. Hence, it is not expected that *ciȩżarów* is understood, but this might not have a negative influence on the overall understanding of the topic of the sentence, which seems to be help understand the target word. However, this also means that while the final CS 3-g contains the whole concept of weightlifting, only *weights* [genitive plural] is part of the final 3-g in PL. It appears as if the correct understanding of the target *siłowniȩ* is supported by correct identification of the concept of sports and the PL keyword *sportowiec* (CS *sportovec*) “sportsman” at the sentence onset, which can result in associative priming. However, it also appears as if neither of the context-aware LMs performed better than the 3-g LMs in reflecting the predictability of the target word.

A relatively low surprisal was assigned to the PL target word *drzewo* in example (4) by the 3-g LMs and by the CS LSTM model, but not by the Transformer and Transformer XL. PL *drzewo* “tree” is a frequent collocate of PL *genealogiczne* “genealogical” just as CS *strom* “tree” is a frequent collocate of CS *genealogický* “genealogical.” In this particular example in which the directly preceding word is a frequent collocate, the 3-g LMs and the LSTM reflect predictability of the target word better than the Transformer LMs.

### 5.4. Controlling for Local Context

We filtered the original dataset (*n* = 149) for sentences for which the 3-g LMs did not reflect predictability of the target word, i.e., sentences with 3-g surprisals above the mean (≥3.2 *Hart* for PL; ≥3.9 *Hart* for CS, cf. Table 5). When we correlate the target word surprisals from the CS context-aware LMs for these sentences (*n* = 78) with the intelligibility of the target words, the correlation of surprisal from the CS LSTM model proves to be higher than the best correlation for the whole set of sentences [*r*(78) = −0.35, *p* < 0.05]. It has to be noted that for the same subset, the correlation did not improve with surprisal from the CS Transformer and the CS Transformer XL. This might suggest that the LSTMs perform somewhat better for such sentences in which the 3-g LMs failed. However, since the difference in correlations is still rather small, this effect could also be due to the lower number of data points and hence the lower number of outliers. Since Jágrová and Avgustinova (2019) found that the lexical distance of the targets is crucial and 3-g surprisal correlates better with the intelligibility of those target words that are not cognates, we also filtered the 78 sentences again for sentences with target words that are not cognates (*n* = 24) and obtained a better correlation with intelligibility and surprisal from the CS LSTM [*r*_(24)_ = −0.457, *p* < 0.05]. The correlations of both filtered subsets are displayed together with the whole dataset for the LSTMs in Figure 4.

**Figure 8:**
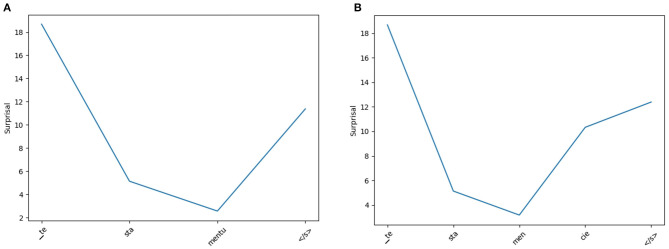
Subword level surprisal of the CS reader model when applied on CS *testamentu* **(A)** and PL *testamencie* **(B)** (both “testament [locative]”). From the perspective of a CS reader, the model displays a rise in surprisal at the unexpected subword *cie* (PL) as opposed to the CS subword units.

While the reader model introduced in section 4.6 did not improve correlation, one can still observe examples in which a change in surprisal on the subword level corresponds to what one would also expect from a CS reader. In Figure 8 this is visualized in an example for the CS and PL locative forms of the word *testament* “testament.” On the CS version of the word, the model trained on CS text has a decreasing surprisal. For the last subword *mentu*, the surprisal is low given the previous subwords *te* and *sta*. On the PL version of the word, the surprisal also decreases for the first three subwords *te, sta*, and *men* as these are shared between CS and PL. For the last subword *cie*, the surprisal increases, however, as this is not the expected ending of this word in CS. We hypothesize that a similar reaction would be evoked in a CS reader. The segmentation into these units can be explained by the fact that *-cie* is a frequent string of characters at the end of CS nominative singular forms of feminine internationalisms, e.g., *policie* “police,” *byrokracie* “bureaucracy,” *Francie* “France.”

## 6. Discussion

We investigated whether surprisal obtained from context-aware LMs correlates better with the intelligibility of highly predictable PL target words to CS readers than surprisal obtained from 3-g LMs in a previous experiment. To this end, we trained seven context-aware LMs on large corpora of PL and CS and scored the stimuli and their CS translations with these models. The surprisal values represent the (un-)predictability of words or their (sub-)sequences in relation to the context.

In general, the correlations of intelligibility and surprisal scores obtained from the context-aware models are slightly higher than the correlations with surprisal from the 3-g LMs. It has to be noted that the differences between these correlations are rather small and the correlations themselves are very low. The highest correlation of intelligibility and surprisal from the LSTMs does not exceed a coefficient of *r* = −0.46, *p* < 0.05 in a number of selected sentences with lexically distant target words, which means that surprisal as an indicator of the predictability of words in context cannot explain more than 21% of the variance in the underlying data. Hence, it has to be noted that target word predictability in context appears to be only one of many other stimulus-related factors (linguistic distance, neighborhood density, associations, interferences from other acquired languages, and divergent grammatical gender) influencing the intelligibility of words in closely related languages in general, not to mention the many possible respondent-related factors that were not elaborated on in this study. Surprisal as a representation of predictability in context does not reach the level of the correlations with the linguistic distance that was many times demonstrated in previous research (e.g., Gooskens, 2007; Vanhove, 2014; Möller and Zeevaert, 2015; Vanhove and Berthele, 2015; Golubović, 2016; Jágrová et al., 2017; Stenger et al., 2017).

In the examples, it appears that the context-aware LMs perform better than 3-g LMs particularly in such sentences, where the helpful part allowing for an association with the correct translation lies outside of the window of two words preceding the target word, i.e., at another position in the sentence than the final 3-g, which is at least in some cases reflected in the lower surprisal scores of the highly predictable target words. However, the 3-g LMs and the LSTMs appear to represent predictability of direct collocates of the target words in the examples better than the context-aware LMs that take more context into account. Nevertheless, the examples discussed in this study were chosen to shed light on the possible processes in the first place and one should not generalize and draw conclusions as to the whole dataset. Also, it has to be noted that the LMs were trained on written language and that human performance in these experiments might be much more influenced by everyday language, which could explain why at least some of the models failed in example (5) and all models failed in example (2) since there might not be many texts about farming or handcraft in the corpora.

We found that the reader model (section 4.6), designed to observe whether cross-lingual similarities can be taken into account with such a type of language model, was only to a certain extent able to predict the greater difficulty of unexpected sequences. This outcome is open for interpretation. It is possible that this model of the reader does not perform ideally, since it also aligns incorrect correspondences, such as ć:č (PL:CS) based on interpretations of the respondents. Consequently, when the CS respondent, for instance, encounters the PL infinitive form *bawić* (*siȩ*) “to play,” the model can approximate that the CS reader will interpret the verb as the noun *bavič* “entertainer,” which is, of course, considered a wrong response in the experiment. The reader model will thus calculate the predictability of *bavič* in the sentence according to the CS model and not the predictability of the correct CS translation of the verb equivalent *hrát* (*si*) “to play.” Nevertheless, it was demonstrated how such a cross-lingual model could work to support a linguistically reasonable model of the reader. Improved modeling of the reader with regard to cross-lingual similarity, also taking linguistic distance into account, could be an interesting avenue for future work. Moreover, predicting the effects of misleading dominant concepts in sentences or interference not only from the L1 of the reader but also from other acquired languages, remains a topic for future research in the field of intercomprehension.

## Data Availability Statement

The original contributions presented in the study are included in the article/Supplementary Material, further inquiries can be directed to the corresponding author/s.

## Author Contributions

KJ initiated this study, gathered and supplied the data from intercomprehension experiments, developed the research question, and performed the analysis of the results (correlations, comparisons, and linguistic interpretations of these). MM and MH developed the language modeling part and ran the technical experiments. TA and DK advised on the project. All authors contributed to the writing.

## Conflict of Interest

The authors declare that the research was conducted in the absence of any commercial or financial relationships that could be construed as a potential conflict of interest.

## Footnotes

^1^http://2018.poleval.pl/index.php/tasks/.

^2^The original stimulus as of Block and Baldwin (2010) uses *spend*.
